# Psychosocial profile of pediatric brain tumor survivors with neurocognitive complaints

**DOI:** 10.1007/s11136-015-1091-7

**Published:** 2015-08-20

**Authors:** Marieke Anna de Ruiter, Antoinette Yvonne Narda Schouten-van Meeteren, Dannis Gilbert van Vuurden, Heleen Maurice-Stam, Corrie Gidding, Laura Rachel Beek, Bernd Granzen, Jaap Oosterlaan, Martha Alexandra Grootenhuis

**Affiliations:** Pediatric Psychosocial Department, Emma Children’s Hospital AMC, Meibergdreef 9, Room A3-241, 1105 AZ Amsterdam, The Netherlands; Department of Pediatric Oncology, Emma Children’s Hospital AMC, Amsterdam, The Netherlands; Department of Pediatrics, VU Medical Center, Amsterdam, The Netherlands; Department of Pediatric Oncology/Hematology, Radboud University Medical Center, Nijmegen, The Netherlands; Department of Medical Psychology, Wilhelmina Children’s Hospital UMC, Utrecht, The Netherlands; Department of Pediatrics, Maastricht University Medical Center, Maastricht, The Netherlands; Department of Clinical Neuropsychology, VU University Amsterdam, Amsterdam, The Netherlands

**Keywords:** Brain tumor, Pediatric oncology, Psychosocial, Late effects of cancer treatment, Quality of life

## Abstract

**Purpose:**

With more children surviving a brain tumor, neurocognitive consequences of the tumor and its treatment become apparent, which could affect psychosocial functioning. The present study therefore aimed to assess psychosocial functioning of pediatric brain tumor survivors (PBTS) in detail.

**Methods:**

Psychosocial functioning of PBTS (8–18 years) with parent-reported neurocognitive complaints was compared to normative data on health-related quality of life (HRQOL), self-esteem, psychosocial adjustment, and executive functioning (one-sample *t* tests) and to a sibling control group on fatigue (independent-samples *t* test). Self-, parent-, and teacher-report questionnaires were included, where appropriate, providing complementary information.

**Results:**

Eighty-two PBTS (mean age 13.4 years, SD 3.2, 49 % males) and 43 healthy siblings (mean age 14.3, SD 2.4, 40 % males) were included. As compared to the normative population, PBTS themselves reported decreased physical, psychological, and generic HRQOL (*d* = 0.39–0.62, *p* < 0.008). Compared to siblings, increased fatigue-related concentration problems (*d* = 0.57, *p* < 0.01) were reported, although self-reported self-esteem and psychosocial adjustment seemed not to be affected. Parents of PBTS reported more psychosocial (*d* = 0.81, *p* < 0.000) and executive problems (*d* = 0.35–0.43, *p* < 0.016) in their child than parents of children in the normative population. Teachers indicated more psychosocial adjustment problems for female PBTS aged 8–11 years than for the female normative population (*d* = 0.69, *p* < 0.025), but they reported no more executive problems.

**Conclusions:**

PBTS with parent-reported neurocognitive complaints showed increased psychosocial problems, as reported by PBTS, parents, and teachers.

**Implications for cancer survivors:**

Systematic screening of psychosocial functioning is necessary so that tailored support from professionals can be offered to PBTS with neurocognitive complaints.

## Introduction

Due to developments in the medical field, survival rates in children with a brain tumor have increased drastically to over 74 % [1]. These successes have led to a growing number of pediatric brain tumor survivors (PBTS). Tumor- and treatment-induced brain injury exerts negative effects on neurocognitive functions, such as attention, processing speed, and memory [2]. As a result, 40–100 % of PBTS suffer from neurocognitive decline [3]. The decline in neurocognitive functioning appears to increase when the children grow older, resulting in an increasing gap between the PBTS and their peers [4–6]. Consequently, children treated for a brain tumor may experience lower academic achievements, resulting in lower vocational success, and decreased psychosocial functioning compared to their healthy peers later in life [7–9].

To date, studies on psychosocial functioning of PBTS are relatively scarce as compared to other types of cancer, especially because children with a brain tumor have often been excluded from studies, due to their atypical outcomes, i.e., they seem to suffer from more serious problems on a variety of domains (e.g., neurocognitive, social, and adjustment problems) than other pediatric cancer survivors [9]. The studies with PBTS found in the literature focused on health-related quality of life (HRQOL), social competence, self-esteem, and fatigue. Attention for HRQOL, a multidimensional construct covering perceived physical, emotional, mental, social, and behavioral components of well-being and functioning [10], in PBTS has started to emerge in the past decades [11]. However, no studies to our knowledge have focused on PBTS with neurocognitive complaints. The results of the studies on HRQOL in PBTS in general were contradictory, with HRQOL comparable to the general population [12], or worse HRQOL in several domains [13]. PBTS reported being bullied, encountering problems with peers, and suffering from stressful and depressive feelings. The researchers mention late effects of the cancer treatment as a possible cause of the decreased HRQOL scores. Decreased neurocognitive functioning was found to be associated with worse HRQOL in PBTS 1 year after treatment [14].

The literature on self-esteem in PBTS is scarce; however, social competence, an aspect of self-esteem which may predict psychosocial functioning, has been investigated in PBTS [15]. In a comprehensive review on social competence, it was concluded that PBTS experienced deficits in this area [16]. In a cross-sectional study, PBTS reported lower social competence than healthy peers and patients with a pediatric brain tumor during treatment, indicative of a decline of social competence of PBTS over time [17]. Furthermore, PBTS experienced more problems with self-confidence and self-esteem compared to leukemia survivors [18].

Fatigue is a common adverse effect of cancer treatment [19, 20]. In addition, due to the nature of their disease, PBTS frequently experience sleep problems and decreased sleep quality, leading to fatigue and negatively influencing daily functioning [21]. Fatigue in childhood cancer survivors and PBTS is associated with worse psychosocial functioning [22, 23].

The influence of executive deficits on psychosocial functioning has been acknowledged [24]. Executive functions, an umbrella term for mental skills concerning planning, behavioral control, and self-regulation, such as attention control, cognitive flexibility, and goal setting are critical skills to function properly in society [24]. Executive functions are often reported to be affected in PBTS [25–27].

Since psychosocial functioning is important but understudied in PBTS as compared to other types of cancer [9, 28], we aimed to investigate various domains of psychosocial functioning of PBTS who suffer from parent-reported neurocognitive complaints: HRQOL, self-esteem, psychosocial adjustment, executive functioning, and fatigue. Based on the previous, we can conclude that it is especially important to study psychosocial functioning of PBTS who suffer from neurocognitive problems, as the literature indicated that patients with neurocognitive problems are vulnerable to psychosocial problems. We take multiple informants (self-, parent-, and teacher report) into account, providing complementary information on how PBTS function, both at home and at school, investigating psychosocial functioning of PBTS who suffer from parent-reported neurocognitive complaints. We hypothesize that PBTS experience decreased HRQOL, self-esteem, psychosocial adjustment, executive functioning, and increased fatigue as reported by PBTS themselves, their parents, and/or teachers.

## Methods

### Procedures and participants

Data collection took place between January 2010 and August 2012, as part of the PRISMA study, a randomized placebo-controlled double-blind trial to investigate whether neurofeedback can improve neurocognitive functioning in PBTS [29]. Eligible for inclusion were children treated for a brain tumor in the Netherlands, aged 8–18 years, who finished treatment >2 years prior to enrollment and who suffered from neurocognitive complaints as reported by a parent on a screening questionnaire, assessing attention, speed, information processing and memory as well as exclusion criteria. Children with a premorbid diagnosis of attention deficit/hyperactivity disorder, a mental or physical condition prohibiting neurocognitive assessment, or insufficient mastery of the Dutch language were excluded from the study.

PBTS (*n* = 249) who were treated in one of the participating Dutch hospitals (Emma Children’s Hospital/Academic Medical Center Amsterdam, VU University Medical Center Amsterdam, University Medical Center Utrecht, St. Radboud University Medical Center, Nijmegen, and University Medical Center Maastricht) and their parents received a letter via their pediatric oncologist or psychologist informing them about the PRISMA study. Additionally, three patients from other hospitals made contact via email about participation, after they learned about the study.

**Fig. 1:**
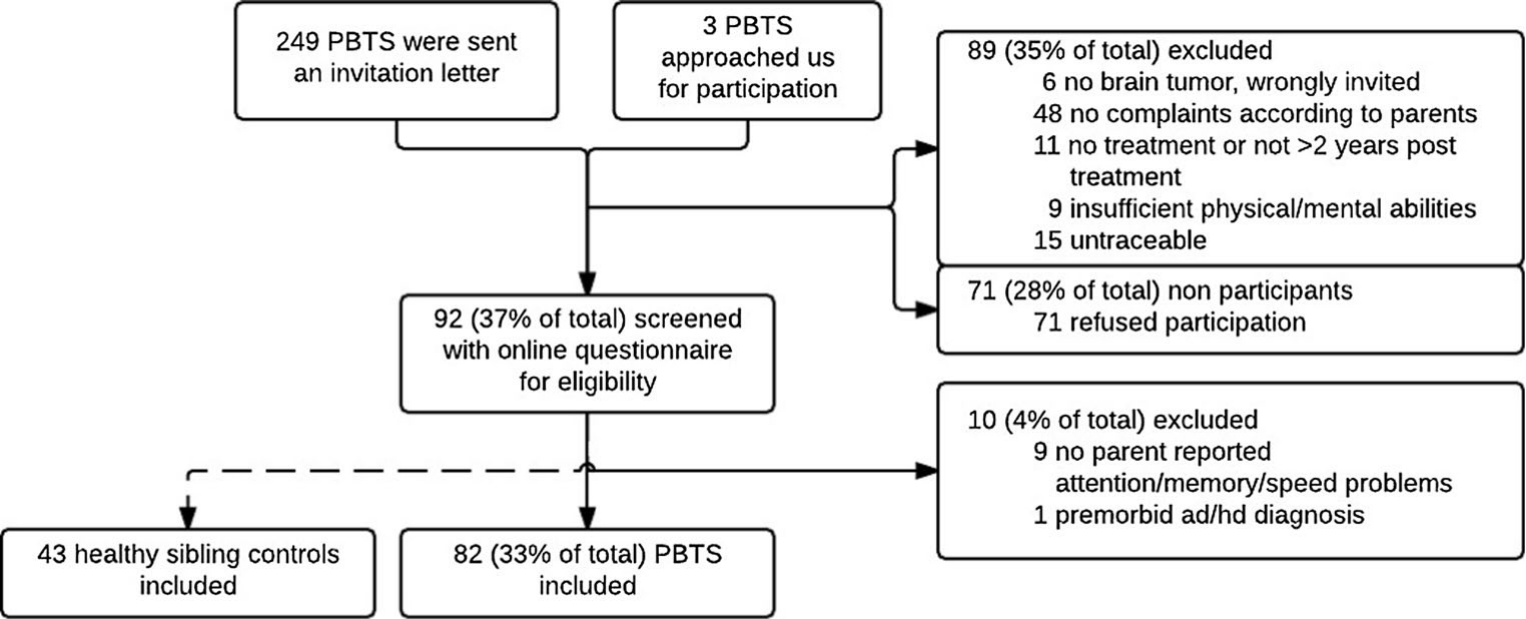
Flowchart of inclusion. *Note* self-reported questionnaires were completed by 81 PBTS and 40 sibling controls. Parent data were available for all 82 PBTS, and teacher data were available for 73 of the included PBTS

Of the PBTS, 89 (35 %) did not meet inclusion criteria and 71 (29 %) declined participation (‘non-participants’) (see Fig. 1 for reasons). Parents of PBTS willing to participate (*n* = 92, 37 %) were provided with an online screening questionnaire concerning their child’s neurocognitive functioning, in order to verify eligibility. Ten PBTS (4 %) were excluded after online screening.

If the included PBTS had a sibling in the age range from 8 to 18 years, he or she was invited via telephone to participate in the control group for the fatigue outcome measure. Siblings were not considered to be optimal as a control group for the other psychosocial outcomes since the cancer diagnosis of the sibling could affect the scores on psychosocial functioning [30, 31].

Informed consent was obtained from the included 82 PBTS (33 %) and 43 siblings. Subsequently, PBTS, parents, siblings, and the case where parents and PBTS gave permission (*n* = 76), teachers of PBTS, were sent the questionnaires via email. For parent-report questionnaires, the primary caregiver was asked to fill out the questionnaire. For the teacher-report questionnaires, the parent was asked to indicate which teacher was most suitable to fill out questionnaires about the functioning of the child.

The study protocol was approved by the Medical Ethics Committee of the Academic Medical Center Amsterdam and was registered at ClinicalTrials.gov (NCT00961922).

### Demographic and medical characteristics

Parents of participating PBTS supplied information on gender and demographics (the parental country of origin and the highest level of parental education). Medical characteristics were taken from the medical records and included tumor histopathology and grade, type of treatment (surgery only vs. chemotherapy and/or radiotherapy with/without surgery), tumor location (supratentorial vs. infratentorial) and prior hydrocephalus, age at diagnosis, and time since diagnosis.

Medical and demographic data were also collected for a subsample of non-participants (45 out of 71 non-participants) to study selection bias at inclusion. As the non-participants declined participation, they were not assessed. To compare the age of participants and non-participants, ‘age at assessment’ for non-participants was calculated as the difference between the birth date and the average assessment date of participating PBTS.

### Outcome measures

It is well-known that proxy report (parent/teacher) on the functioning of chronically ill children often yields discrepancies with self-report, although results of studies have been contradictory [32]. For this reason, we included a combination of self-report, parent-report, and teacher-report questionnaires.

#### Self-report

##### HRQOL

The Kidscreen-27 was administered to evaluate HRQOL in children by means of 27 items, scored on a 5-point Likert scale, divided over 5 dimensions: physical well-being, psychological well-being, autonomy and parents, peers and social support, and school environment [33]. In addition, a Generic score was calculated by summing the ten items that comprise the Kidscreen-10, a shorter version of the Kidscreen, derived from the Kidscreen-27 [34]. Raw scores for each dimension were transformed into *T* values with a mean of 50 and a standard deviation of 10 in a European sample.

*T* values of a Dutch normative sample are available. Higher scores indicated better HRQOL. The Kidscreen-27 and Kidscreen-10 have good levels of validity and reliability (Cronbach’s alpha normative samples >0.70; Cronbach’s alpha PBTS 0.71–0.88) [33, 34]. The Dutch normative sample did not differ in age and gender distribution from the PBTS group (*p* > 0.10).

##### Self-esteem

We used the self-perception profile for children (SPPC, age 8–12) and adolescents (SPPA, age 12–18) to investigate self-esteem [35–37]. The SPPC consists of 36 items, divided into six scales: scholastic competence, social acceptance, athletic competence, physical appearance, behavioral conduct, and global self-worth. The adolescent version (SPPA) contains 35 items and comprises one additional scale: close friendship. Each item was presented on a 4-point Likert scale, with higher scores indicating stronger self-esteem. The SPPC and SPPA have acceptable to good validity and reliability (Cronbach’s alpha Dutch manual >0.70; Cronbach’s alpha PBTS 0.62–0.91) [36, 37]. The manual provided mean scores for males and females separately. For comparison with the total group of PBTS, scores of males and females in the normative population were combined after weighting by the gender distribution in the PBTS group. Age was not taken into account, as the SPPC and SPPA have separate norms based on age.

##### Psychosocial adjustment

The Strengths and Difficulties Questionnaire (SDQ) was used to assess psychosocial adjustment [38, 39]. The items were scored on a 3-point Likert scale. A total problem score was calculated by adding the scores of 20 items, with a higher score indicating more problems. The SDQ total problem score has good validity and reliability (Cronbach’s alpha Dutch controls = 0.70; Cronbach’s alpha PBTS = 0.77) [39]. Dutch norms were available for children aged 11–16 years; therefore, analyses on the SDQ were restricted to PBTS aged 11–16 years. The gender distribution did not differ between the Dutch normative group and the PBTS, but mean age of the normative population was lower than of the PBTS. However, since total problem score is not affected by age, age was not taken into account in the analysis.

##### Fatigue

Fatigue was measured with the checklist individual strength (CIS) [40]. The CIS is a questionnaire that measures fatigue-related problems and consists of 20 items, scored on a 7-point Likert scale. The four subscales were subjective fatigue, concentration, motivation, and physical activity. A total score was calculated by summing up all items. Higher scores indicated more fatigue-related problems. The CIS has good reliability, with Cronbach’s alpha of the sibling controls and the PBTS 0.72–0.94. The data collected in the sibling control group were used for comparison with the PBTS. The sibling control group and the PBTS did not differ significantly (*p* > 0.10) in gender and age.

#### Parent report

##### Psychosocial adjustment

The SDQ was used to measure the parental perspective of PBTS’ psychosocial adjustment (see ‘Self-report’ for the description of the questionnaire). Reliability of the total problem score is good (Cronbach’s alpha Dutch controls = 0.70; Cronbach’s alpha PBTS = 0.77) [39]. PBTS were compared to a Dutch normative sample of children aged 8–16. The Dutch normative sample did not differ in age and gender from the PBTS (*p* > 0.10).

##### Executive functioning

Parents rated their child’s behavioral executive functioning using the behavior rating inventory of executive function (BRIEF) [41]. The BRIEF contains 75 items, scored on a 3-point Likert scale. The scores were summarized in eight scales (inhibit, shift, emotional control, initiate, working memory, plan/organize, organization of materials, and monitor), two indices (behavioral index and metacognition index), and a total score. The raw scores of the scales and indices were transformed into age- and gender-specific standardized *T* scores, as provided in the manual, with a mean of 50 and a standard deviation of 10. Higher scores indicated more problems. Validity and reliability range from good to excellent, with Cronbach’s alphas reported in the manual between 0.78 and 0.96 and Cronbach’s alphas of the PBTS between 0.66 and 0.94 [42].

#### Teacher report

##### Psychosocial adjustment

The SDQ was used to measure the teacher perspective of psychosocial adjustment of the PBTS (see ‘Self-report’ for the description of the questionnaire). The reliability of the total problem score of the teacher report is reported to be good (Cronbach’s alpha Dutch controls = 0.88; Cronbach’s alpha PBTS = 0.77) [39]. A Dutch normative population of children aged 8–11 was available; therefore, the answers from teachers of PBTS within that age range were analyzed. The total problem score was analyzed separately for females and males, because the PBTS sample had more females than the normative population. The mean age of the Dutch normative population did not differ from the mean age of the PBTS aged 8–11.

##### Executive functioning

The BRIEF teacher-report version measures executive functioning of PBTS in the school situation (see ‘Parent-report’ for the description of the BRIEF). Validity and reliability are good to excellent, with Cronbach’s alphas ranging from 0.88 to 0.98 as reported in the manual and between 0.82 and 0.97 of the PBTS [42].

### Statistical analyses

All analyses were conducted using SPSS version 20.0 (SPSS Inc., Chicago, IL, USA). To be able to detect possible confounders, one-sample *t* tests (age) and binomial tests (gender) were performed to test differences between PBTS and the normative population. Independent-samples *t* tests (age) and Chi-square tests (gender, country of birth, education) were used to test differences between PBTS and sibling controls. A *p* value of <0.10 was considered statistically significant for these analyses.

Differences between the participating PBTS and the subsample of 45 non-participating PBTS were tested with one-sample *t* tests (age at assessment, age at diagnosis, time since diagnosis), binomial tests (gender, tumor grade, tumor location, treatment, and hydrocephalus), or Chi-square test (tumor type).

One-sample *t* tests were used to evaluate differences between PBTS and the normative population regarding self-reported HRQOL, self-esteem, and regarding psychosocial adjustment, and proxy-reported psychosocial adjustment and executive functioning. Self-reported fatigue was analyzed with independent-samples *t* test (PBTS vs. sibling controls).

Effect sizes were calculated in terms of Cohen’s *d,* with 0.20, 0.50, and 0.80, reflecting small, medium, and large effect sizes, respectively [43]. To adjust for multiple testing, Bonferroni correction was applied to the significance levels, as follows: HRQOL and self-esteem 8–11 years 0.05/6 = 0.008; self-esteem 12–18 years 0.05/7 = 0.007; fatigue 0.05/5 = 0.01; indices/total executive functioning 0.05/3 = 0.016; scales executive functioning 0.05/8 = 0.006. Differences with *p* values <0.05 in combination with effect size >0.35 were considered to be trends.

## Results

### Participants

The inclusion flowchart is depicted in Fig. 1. One enrolled PBTS, three enrolled siblings, and three teachers of enrolled PBTS did not complete the questionnaires. Self-report data were therefore available for 81 PBTS, 40 siblings, and teacher-report data for 73 PBTS.

Characteristics of the participating PBTS, the sibling control group and the non-participating PBTS are depicted in Table 1. Regarding the demographics, 20 of the participating PBTS received special education (24 %) and 39 have been held back a class (48 %). The participating and non-participating PBTS were comparable in age at assessment, gender, and education (*p* > 0.062). Participants and non-participants did not differ in tumor location, but they did differ with regard to the distribution of tumor grade, with more high-grade tumors in the participants than in the non-participants (*p* < 0.05). The participants were younger at diagnosis (*p* < 0.05) and had a longer interval past diagnosis than the non-participants (*p* < 0.05). More participating than non-participating PBTS underwent radiotherapy (*p* < 0.05) and chemotherapy (*p* = 0.001). The participating PBTS and the sibling control group were comparable in age, gender, parental country of origin, and the highest level of parental education (*p* > 0.324).

**Table 1:** Demographics and medical information of participating pediatric brain tumor survivors, sibling controls, and non-participating pediatric brain tumor survivors

	PBTS participants		Controls		PBTS non-participants	
	*n* = 82		*n* = 43		*n* = 45	
	*M*	SD	*M*	SD	*M*	SD
Age						
Age at assessment	13.85	3.15	14.27	2.44	14.28	3.04
Age diagnosis	6.87	3.77	–	–	8.23	3.95
Time since diagnosis	6.98	3.57	–	–	6.05	3.31

	*n*	%	*n*	%	*n*	%
Gender						
Boys	40	49	17	40	26	58
Country of origin mother						
Netherlands	71	87	37	86	n/a	n/a
Other	11	13	9	14	n/a	n/a
Country of origin father						
Netherlands	73	89	40	93	n/a	n/a
Other	9	11	3	7	n/a	n/a
Highest education parent^a^						
Low or Intermediate	39	48	23	54	n/a	n/a
High	43	52	20	46	n/a	n/a
Education						
Regular education	62	80	n/a	n/a	28	62
Special education	20	20	n/a	n/a	11	24
Unknown	0	0	n/a	n/a	6	13
Tumor type and grade						
High grade	34	42	–	–	13	29
Medulloblastoma	12	15	–	–	6	13
Supratentorial PNET	8	10	–	–	2	4
Ependymoma	5	6	–	–	2	4
Astrocytoma gr III	5	6	–	–	1	2
Germ cell tumor	4	5	–	–	2	4
Low grade	48	59	–	–	32	71
Low grade glioma	35	43	–	–	26	58
Craniopharyngioma	7	9	–	–	5	11
Plexus papilloma	6	7	–	–	1	2
Treatment						
Radiotherapy	34	42	–	–	14	31
Chemotherapy	35	43	–	–	12	27
Surgery^b^	72	88	–	–	41	91
Other	2	2	–	–	1	2
Biopsy only	1	1	–	–	1	2
CSF pressure relief only	1	1	–	–	0	0
Location						
Supratentorial	46	56	–	–	22	49
Infratentorial	36	44	–	–	23	51
Hydrocephalus						
Yes	39	48	–	–	n/a	n/a
No	43	52	–	–	n/a	n/a

The information was available for 45 of 71 non-participanting PBTS. The siblings did not differ significantly from the participating PBTS on any of the variables. The non-participanting PBTS differed from the participants on age at diagnosis, time since diagnosis, tumor type, tumor grade, radiotherapy and chemotherapy

*PBTS* pediatric brain tumor survivors,* M* mean,* SD* standard deviation,* n/a* not available

* *p* < .05; ** *p* < .001

^a^Highest education of father or mother is reported: Low or Intermediate = Primary education, general secondary education and secondary vocational education; High = Higher vocational education and university

^b^37 patients were treated with surgery only

### Outcomes

**Fig. 2:**
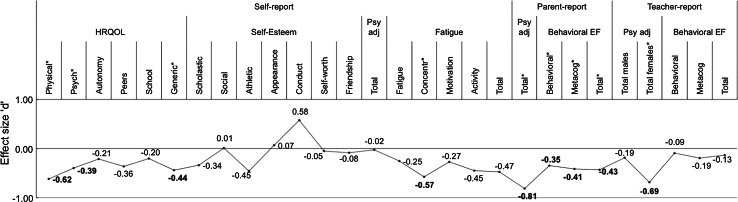
Profile of psychosocial functioning in pediatric brain tumor survivors in standardized effect sizes as compared to the mean of the control group (0.00). *Significant difference between PBTS and controls after the Bonferroni correction, effect sizes presented in *red* and *bold*. *Note*. Effect sizes ‘d’ were calculated using the pooled standard deviation. Scores have been adjusted in a way that for all domains, lower scores reflect worse psychosocial functioning. For self-esteem, weighted average effect sizes of the SPPC and SPPA are depicted. For teacher report of psychosocial adjustment (SDQ), scores for males and females are reported separately due to more females in our sample as compared to the control group. HRQOL = health-related quality of life, physical = physical well-being subscale of the Kidscreen-27, psych = psychological well-being subscale of the Kidscreen-27, autonomy = autonomy and parents subscale of the Kidscreen-27, peers = peers and social support subscale of the Kidscreen-27, school = school environment subscale of the Kidscreen-27, generic = generic health-related quality of life subscale of the Kidscreen-10, scholastic = scholastic competence subscale of the SPPC/SPPA, social = social acceptance subscale of the SPPC/SPPA, athletic = athletic competence subscale of the SPPC/SPPA, appearance = physical appearance subscale of the SPPC/SPPA, conduct = behavioral conduct subscale of the SPPC/SPPA, self-worth = global self-worth subscale of the SPPC/SPPA, friendship = close friendship subscale of the SPPA, psy adj = psychosocial adjustment, total score SDQ, fatigue = subjective fatigue subscale of the CIS, concentr = concentration subscale of the CIS, motivation = motivation subscale of the CIS, activity = physical activity subscale of the CIS, behavioral EF = behavioral executive functioning BRIEF, behavioral = behavioral regulation index of the BRIEF, metacog = metacognition index of the BRIEF. (Color figure online)

In Tables 2 and 3, the results of the self-report and proxy-report questionnaires’ analyses are displayed, respectively. Figure 2 is a graphical summary of the results (effect sizes) from Tables 2 and 3, showing the profile of psychosocial functioning in PBTS. For self-esteem, average effect sizes are depicted, weighted by the number of PBTS who completed the SPPC and SPPA.

**Table 2:** Psychosocial functioning of the pediatric brain tumor survivors compared to the controls; self-report

Measure	*n*	PBTS		Controls		Group differences	
		*M*	SD	*M*	SD	*d*	*p*
HRQOL—KIDSCREEN-2 [35]/Kidscreen-10 [36]							
Physical well-being	81	46.69	9.69	52.88	10.02	0.62	**<0.001**
Psychological well-being	81	49.09	9.09	52.79	9.46	0.39	**<0.001**
Autonomy and parents	81	51.99	8.21	53.95	9.51	0.21	0.035
Peers and social support	81	49.11	10.94	52.36	9.04	0.36	0.009
School environment	78	51.08	8.70	53.06	9.71	0.20	0.049
Generic (Kidscreen-10)	78	49.55	8.32	54.10	10.40	0.44	**<0.001**
Self-esteem—SPPC (8–12) [37]							
Scholastic competence	24	14.79	3.79	16.60	3.46	0.52	0.028
Social acceptance	24	18.33	3.91	17.55	3.68	0.21	0.334
Athletic competence	24	16.08	4.24	17.88	3.24	0.56	0.049
Physical appearance	24	20.67	3.50	19.24	3.95	0.36	0.058
Behavioral conduct	24	20.29	3.58	17.72	2.85	0.91	**0.002**
Global self-worth	24	20.63	3.32	19.55	3.06	0.35	0.126
Self-esteem—SPPA (12–18) [38]							
Scholastic competence	57	13.23	2.93	13.88	2.51	0.26	0.097
Social acceptance	57	15.14	2.97	15.34	2.73	0.07	0.607
Athletic competence	57	12.37	4.26	13.74	3.35	0.41	0.018
Physical appearance	57	13.67	3.50	13.82	3.27	0.05	0.742
Behavioral conduct	57	15.49	3.67	14.24	2.88	0.44	0.013
Global self-worth	57	16.47	3.52	17.08	2.80	0.22	0.196
Close friendship	57	15.28	3.71	15.52	2.84	0.08	0.634
Psychosocial adjustment—SDQ (11–16) [39]							
Total problem score	48	10.02	5.09	9.90	4.90	0.02	0.870
Fatigue—CIS [41]^a^							
Subjective fatigue	76	23.57	11.16	20.53	10.75	0.25	0.168
Concentration	76	19.09	7.78	14.45	7.19	0.57	**0.003**
Motivation	76	11.29	4.91	9.82	4.87	0.27	0.133
Physical activity	76	9.27	5.00	6.97	4.16	0.45	0.011
Total score	76	63.23	21.80	51.76	21.88	0.47	0.010

Significant differences after Bonferroni correction are presented in bold. Effect sizes ‘d’ were calculated by dividing the difference in mean score between the PBTS and the normative population or sibling controls by the pooled standard deviation. Lower scores reflect worse HRQOL and Self-Esteem. Higher scors reflect more problems on Psychosocial adjustment and fatigue

*PBTS* pediatric brain tumor survivors,* HRQOL* health related quality of life,* M* mean,* SD* standard deviation

^ a^Sibling controls.* n* = 40

**Table 3:** Psychosocial functioning of the pediatric brain tumor survivors compared to the controls; proxy report

	*n*	PBTS		Controls		Group differences	
		*M*	SD	*M*	SD	*d*	*p*
*Parent report*							
Psychosocial adjustment—SDQ (8–16) [39]							
Total problem score	67	11.01	5.16	6.70	5.30	0.81	**<0.001**
Behavioral executive functioning—BRIEF [43]							
Behavioral index	82	53.48	11.22	50.00	10.00	0.35	**0.002**
Metacognition index	82	54.09	8.11	50.00	10.00	0.41	**<0.001**
Total score	82	54.29	8.45	50.00	10.00	0.43	**<0.001**
*Teacher report*							
Psychosocial adjustment—SDQ (8–12) [39]							
Total problem score males	9	10.33	9.44	9.10	6.60	0.19	0.582
Total problem score females	21	9.71	5.60	5.80	5.70	0.69	**0.004**
Behavioral executive functioning—BRIEF [43]							
Behavioral index	73	50.89	12.41	50.00	10.00	0.09	0.511
Metacognition index	73	51.89	13.90	50.00	10.00	0.19	0.249
Total score	73	51.29	12.72	50.00	10.00	0.13	0.395

Significant differences after Bonferroni correction are presented in bold. Effect sizes ‘d’ were calculated by dividing the difference in mean score between the PBTS and the normative population by the pooled standard deviation. Higher scores reflect worse psychosocial adjustment and behavioral functioning

*PBTS* pediatric brain tumor survivors, *M* mean, *SD* standard deviation

#### Self-report

##### HRQOL

PBTS showed significantly worse HRQOL than the normative sample (*p* < 0.008) on 2 subscales of the Kidscreen-27: physical well-being and psychological well-being, and on the generic scale (medium-to-large effect sizes). A tendency toward lower HRQOL in PBTS than the norm was found for peers and social support (*p* < 0.05; medium effect size).

##### Self-esteem

PBTS aged 8–11 years obtained significantly higher behavioral conduct scores compared to the normative population (*p* < 0.008, large effect size), indicating higher self-esteem regarding their behavior; PBTS aged 12–18 tended toward higher self-esteem on this scale (*p* < 0.05, medium effect size). A trend toward lower self-esteem in PBTS aged 8–11 was found for scholastic competence and for athletic competence, and also for athletic competence of PBTS aged 12–18 (*p* < 0.05, medium effect size). No differences between the normative population and the PBTS were observed on the other scales.

##### Psychosocial adjustment

PBTS between 11 and 16 years of age did not experience more psychosocial adjustment problems than the normative population as shown by their total problem score of the SDQ.

##### Fatigue

PBTS reported more concentration problems than the sibling control group (*p* < 0.01, medium effect size). A trend toward decreased physical activity in PBTS compared to the sibling control group was found as well as a trend toward a higher total scale compared to the siblings (*p* < 0.05, medium effect sizes), indicating more fatigue-related problems. The PBTS did not differ from the siblings on subjective fatigue and motivation problems.

#### Parent report

##### Psychosocial adjustment

The parent-reported total problem score of psychosocial adjustment (SDQ) was higher in the PBTS than in the norm (*p* < 0.001, large effect size), indicating more problems in psychosocial adjustment.

##### Executive functioning

Parents of PBTS considered their children’s behavioral expressions of executive functioning to be significantly worse than parents in the normative population. More specifically, PBTS had lower scores regarding the two indices and the total score (*p* < 0.016; medium effect sizes). Subsequent analyses showed worse functioning on the scales’ emotional control, and on initiate and working memory (*p* < 0.006; *d*s 0.47, 0.59, and 0.71, respectively). No significant differences were found on the other subscales.

#### Teacher report

##### Psychosocial adjustment

For the female PBTS aged 8–11 years (*n* = 9), teachers reported significantly higher total problem scores (SDQ) than the norm (*p* < 0.01, large effect size). No difference was found between the male PBTS (*n* = 21) and the males in the normative population.

##### Executive functioning

According to the teacher report, no differences were found between the PBTS and the normative population on the indices and the total score of the BRIEF.

## Discussion

This study provides the first multidimensional (self-, parent- and teacher report) view of psychosocial functioning of PBTS with parent-reported neurocognitive complaints. The multidimensional approach is an advantage of the study because of the symptom burden of patients and complexity of their social situation. PBTS showed decreased psychosocial functioning on a number of the tested domains: self-reported HRQOL and fatigue, parent-reported psychosocial adjustment and executive functioning, and teacher-reported psychosocial adjustment for females only. These results are in line with a study by Meeske et al. [20], who reported PBTS to exhibit problems in physical, social, psychosocial, school, cognitive domains, and fatigue. The decreased HRQOL scores of PBTS on psychological well-being may be caused by the neurocognitive complaints from which they suffer. This is supported by the trend we found toward lower self-esteem regarding scholastic competence the PBTS show and by the literature [13]. This should be further studied in future studies. However, despite the neurocognitive complaints, PBTS functioned within normal ranges in several psychosocial domains or showed only trends toward worse functioning: self-reported self-esteem and psychosocial adjustment, and teacher-reported executive functioning and psychosocial adjustment for males.

Physical functioning was specifically compromised in PBTS. Besides worse physical HRQOL, a tendency toward decreased self-reported athletic competence (domain of self-esteem) and decreased physical activity (domain of the fatigue questionnaire) was observed. It is known that PBTS are at increased risk of functional impairments, which is related to physical self-esteem [44]. It is important that professionals working with PBTS are aware of these possible late effects and monitor physical well-being in relation to self-esteem and HRQOL.

Regarding self-esteem, no problems other than the trends toward physical-related and scholastic-related problems were seen. PBTS behavioral conduct scores were even better than the norm. This positive finding has been observed previously, e.g., after a trauma [45], but has recently also received attention in pediatric oncology literature [46]. It has been attributed, among other factors, to the resilience of the PBTS and posttraumatic growth.

The PBTS in our sample did not report more psychosocial adjustment problems as assessed with the SDQ than their peers, in contrast with parents and teachers who did report psychosocial adjustment problems in PBTS. This finding is not surprising, as in the literature it has been found that both healthy children and childhood cancer survivors typically report different levels of psychosocial problems than their parents and/or teachers [47]. Some studies found child-reported levels of problems to be higher than parent- and/or teacher-reported levels [48], while other studies found the opposite [49]. Sato et al. [50] concluded that parent and child ratings are influenced by different factors. Among others, parents’ perception was influenced by their level of distress, whereas the child’s perception tended to be dependent on trait anxiety. Others found that childhood cancer survivors may report less psychosocial problems influenced by social desirability, stress-related growth, or a positive coping strategy [51, 52]. In this study, the diagnosis and treatment resulting neurocognitive consequences might have led to increased parental distress, causing parents to report more problems than their children. Another possible reason for the discrepancy between the observed scores of the different informants in our study is the age difference in self-reports versus parent and teacher reports concerning psychosocial adjustment: Due to age-restricted normative data, the results on the self-reported SDQ were based on PBTS aged 11–16 (*n* = 48), whereas parent- and teacher-reported results were based on PBTS aged 8–16 (*n* = 67) and 8–12 (*n* = 30), respectively.

Teachers reported no executive problems in PBTS, whereas parents did, especially regarding emotional control, initiation, and working memory. This discrepancy could be the result of the ‘observation environment.’ Teachers observed the PBTS in a school environment, which is more structured than the home situation. Possibly, the problems parents saw at home did not exist in the same way in structured settings like school. Turner and colleagues describe problems of PBTS to increase as they leave the structured school environment [53]. This implies that PBTS may benefit from a structured environment. Another reason for the difference between the parent and teacher perspective could be that they have a different reference background. Parents know the child’s premorbid functioning, whereas teachers have the behavior of classmates as a reference. A large proportion of the children in our sample were in special education (24 %), where many classmates suffered from chronic conditions, which could also affect psychosocial functioning [54].

This study has some limitations to take into account. The results are not generalizable to the PBTS population as a whole, since PBTS in this study were selected on the basis of parent-reported neurocognitive problems and the willingness to participate in a study of a treatment aimed to improve neurocognitive functioning. This may have led to an overestimation of the psychosocial problems. It is easy to consider this a non-representative sample, but we have to take into account that many children with a brain tumor suffer from neurocognitive problems (40–100 %). Therefore, this study sheds light on a vulnerable group of PBTS. Awareness for their psychosocial functioning from a complementary perspective is of utmost importance. Another limitation of the study is that normative data were not available for all questionnaires within all age groups. So for some outcomes, especially the SDQ, comparison with the normative population was possible for only small subgroups of PBTS. This limits the reliability and generalizability of the results. For this reason, we would like to urge future studies to aim at collecting norm data for broader age ranges. Nevertheless, this study adds to the existing knowledge as it provides a broad, multidimensional profile of functioning of PBTS with neurocognitive complaints, based on multiinformants.

Better insight into psychosocial functioning in the growing group of PBTS with neurocognitive complaints will help professionals to identify those patients susceptible to developing psychosocial problems. Timely identification is important to prevent problems from escalating. Screening for possible psychosocial late effects should be done in a systematic way, preferably by using the perspective of the patient, parent, and teacher. In daily clinical practice, patient- and/or parent-reported outcomes (PROs) are recommended, because this will increase awareness of and attention for psychosocial functioning during routine checkups. Increased awareness can improve provision of aftercare [55]. Furthermore, providing tailored support to this group of vulnerable children is necessary. Interventions for PBTS with (imminent) psychosocial problems should be aimed at improving HRQOL, coping with fatigue, and providing structure in daily life.
